# Prehospital acute traumatic pain assessment and management practices in the Western Cape, South Africa: a retrospective review

**DOI:** 10.1186/s12245-020-00278-w

**Published:** 2020-05-05

**Authors:** Andrit Lourens, Romy Parker, Peter Hodkinson

**Affiliations:** 1 Division of Emergency Medicine, University of Cape Town (UCT), Cape Town, South Africa; 2 Department of Anaesthesia and Perioperative Medicine, University of Cape Town (UCT), Cape Town, South Africa

**Keywords:** Prehospital, Acute pain assessment and management, Analgesia, Trauma

## Abstract

**Background:**

Trauma is a common aetiology of acute pain in the emergency setting, and traumatic injuries have been recognised as a global public health crisis leading to numerous deaths and disabilities. This study aimed to identify the prevalence of acute pain among high acuity trauma patients presenting to a public sector emergency medical service and to describe prehospital acute traumatic pain assessment and management practices amongst emergency care providers in the Western Cape Province, South Africa.

**Methods:**

A retrospective review of electronic patient care reports of trauma patients treated by the South African Western Cape Emergency Medical Services between January 1 and December 31, 2017 was conducted. Stratified random sampling was utilised to select 2401 trauma patients out of 24,575 that met the inclusion criteria.

**Results:**

Of the 2401 patients reviewed, 435 (18.1%) had a pain score recorded, of which 423 (97.2%) were experiencing pain. An additional 8.1% (*n* = 194) of patients had pain or tenderness mentioned in the working diagnosis but no pain score noted. Eighty-one (18.6%) patients experienced mild pain, 175 (40.2%) moderate pain and 167 (38.2%) severe pain. No association was found between a pain score recorded and age group (≤ 14 versus > 14 years) (*p* = 0.649) or gender (*p* = 0.139). Only 7.6% of patients with moderate-to-severe pain and 2.8% of all trauma patients received any form of analgesic medication. No association was found between the administration of analgesia and age group (≤ 14 versus > 14 years) (*p* = 0.151) or gender (*p* = 0.054). Patients were more likely to receive analgesia if they had a pain score recorded (*p* < 0.001), were managed by advanced life support practitioners (*p* < 0.001) or had severe pain (*p* = 0.001).

**Conclusion:**

Acute trauma pain assessment and management practices in this prehospital cohort are less well established than reported elsewhere and whether this reflects emergency care training, institutional culture, scopes of practice or analgesic resources, requires further research. Emergency medical services need to monitor and promote quality pain care, enhance pain education and ensure that all levels of emergency care providers have access to analgesic medication approved for prehospital use. Clear and rational guidelines would enable better pain management by all cadres of providers, for all levels of pain.

## Background

Traumatic injuries are a global public health crisis with more than 4.8 million deaths annually, and many more left disabled [1, 2]. In South Africa, the high burden of trauma is evidenced by death rates secondary to interpersonal violence/homicide and road traffic accidents, far higher than the global rate [3]. Many studies identify traumatic injuries as the foremost aetiology of acute pain in the prehospital [4–8] and emergency department (ED) settings [9, 10], and patients with acute trauma regularly experience moderate-to-severe pain [5, 9, 11, 12] which is likely to be more widespread in severely injured or high acuity trauma patients.

In addition to relieving suffering and enabling diagnostic and treatment processes in the acute setting, pain control carries further benefits which include reducing the psychological (e.g. anxiety) and physiological effects of acute pain, infection risk, the risk for developing chronic pain and improving patient satisfaction, recovery time and outcomes [13–15]. Failing to adequately manage acute pain may contribute to continued impaired physical function and the subsequent development of psychological disorders (such as depression) and reduced quality of life [13, 14, 16]. Although a fundamental aspect of prehospital emergency care [17], the poor quality of acute pain assessment and management, for any aetiology, in the prehospital arena remains a concern worldwide [5–7, 18, 19].

In the African prehospital setting, little is known about acute pain, with no studies reporting on the epidemiological characteristics of acute traumatic pain, and limited studies describing pain management practices [20, 21]. The paucity of data has been identified as one of many obstacles limiting the advancement of the field of prehospital emergency care in the African region [22]. The aim of this study was to identify the prevalence of acute pain among high acuity trauma patients and to describe prehospital acute traumatic pain assessment and management practices amongst emergency care providers in the Western Cape, South Africa.

## Methods

A retrospective review of electronic patient care reports (ePCRs) of high acuity trauma patients treated by the Western Cape Emergency Medical Services (WCEMS) was conducted between January 1 and December 31, 2017. The WCEMS is a government-operated emergency medical service (EMS) which serves the communities of the Western Cape, one of the nine South African provinces, with an area of 190,370 km^2^ and a population exceeding 6.3 million. WCEMS operates around 250 ambulances throughout the province, staffed at either basic life support (BLS), intermediate life support (ILS) or advanced life support (ALS) emergency care levels.

Prehospital emergency care education in South Africa has occurred through short course (three-tiered) training, but increasingly through higher education and training [23, 24]. Most ambulances are staffed by ILS and BLS practitioners [24], who can request assistance from a higher qualified practitioner (if available). The extent of pain education is hard to gauge and likely varies between training institutions across South Africa.

BLS and ILS practitioners are restricted to the use of self-administered inhaled nitrous oxide (Entonox®) for the relief of pain arising from myocardial infarction, musculoskeletal trauma, burns, active labour and any other condition requiring pain relief where no contraindication is present. ALS practitioners, according to their specific qualifications, may administer intravenous (IV) morphine (some requiring permission from university degree ALS practitioners or a doctor), and IV or intranasal (IN) ketamine may be administered by ALS practitioners with a university degree.

**Fig. 1:**
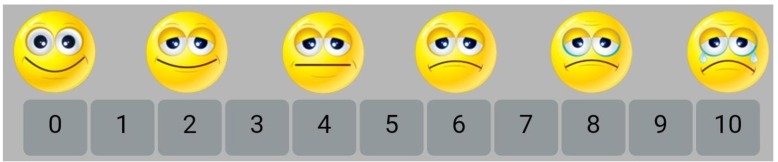
Pain assessment tool using smiley emoticons [26]

In 2016, WCEMS rolled out an ePCR system which replaced paper-based patient care reports with real-time digital capturing of patient care records of all prehospital patient encounters [25]. The system incorporates a pain assessment tool (see Fig. 1) similar to the Wong-Baker Faces scale, rating pain between 0 (no pain) and 10 (worst pain imaginable) with six smiley emoticons [personal communication R Booley, 07/2019]. The system also allows for recording pain characteristics (onset, quality, provoking/palliating factors and radiation) and updated pain scores.

Inclusion criteria were adult and paediatric patients with acute trauma (primary emergencies) and a South African Triage Scale (SATS) final priority colour of yellow, orange or red which denote urgent, very urgent or emergency patients, respectively [27], managed in the prehospital setting by emergency care providers in the Western Cape, South Africa, in 2017. Based on the National Department of Health 2012 age definitions for South Africa, paediatric was defined as patients’ ≤ 14 years. Medical patients, interfacility transfers and patients with a green (non-urgent) or blue (deceased) final priority colour were excluded. The SATS is a triage tool used to measure patient acuity in the South African context, and although developed and validated in the hospital setting, it is also widely used prehospitally to guide optimal disposition (patient destination) decisions [28, 29].

**Fig. 2:**
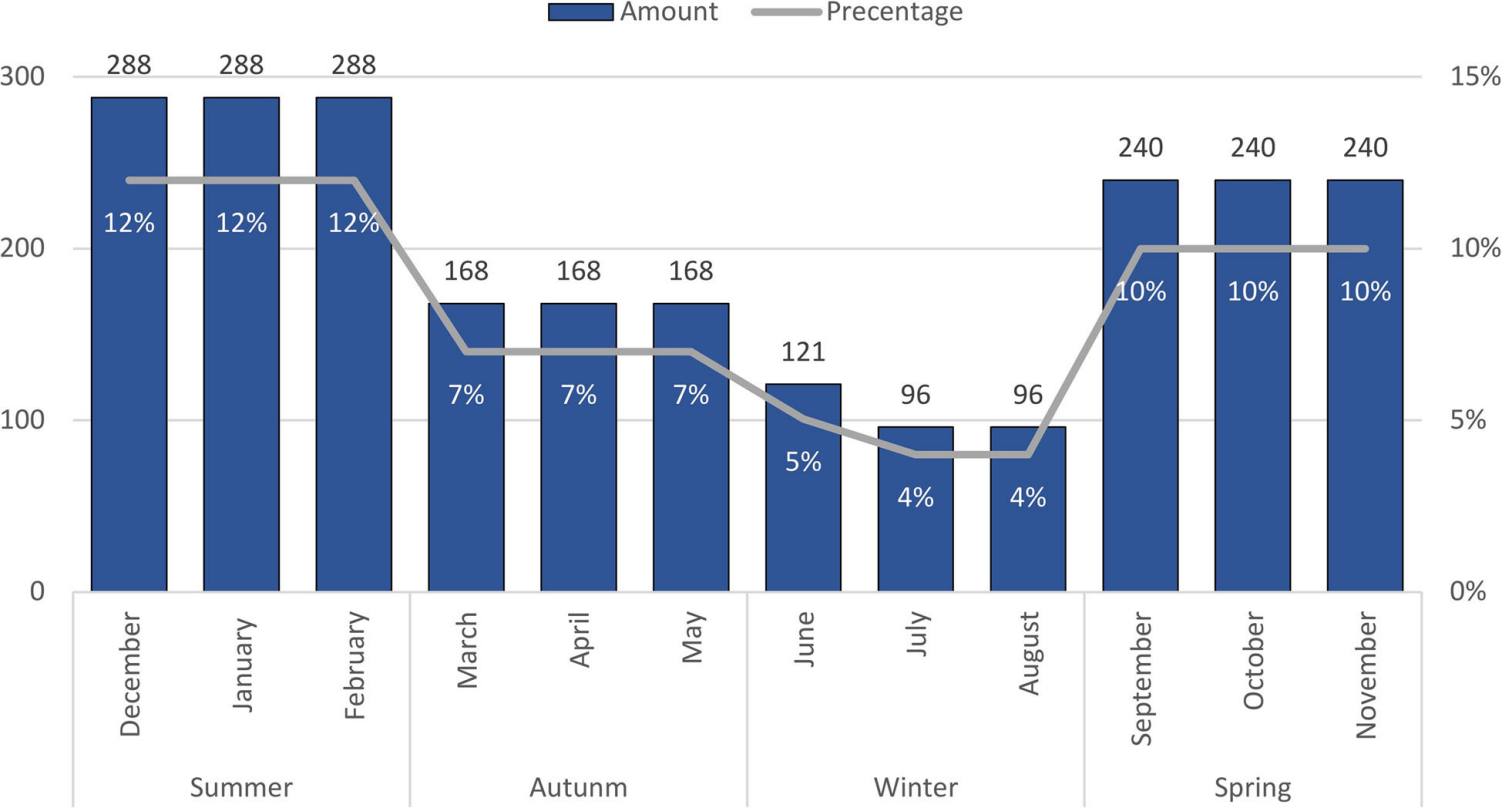
Number of patients randomly sampled per month

A total of 24,575 trauma patients met the inclusion criteria. Stratified random sampling was utilised to select a representative sample of the study population. A sample of 2401 was calculated using an online sample size calculator [30] with an estimated acute traumatic pain prevalence of 50%, 2% precision, 95% confidence interval and an infinite population. Acute traumatic injury prevalence is thought to vary during the year; resultantly, the sample was stratified per month (Fig. 2) to adjust for possible seasonal variation. Two thirds (66%) of data were selected during spring (30%) and summer (36%) including the December/January festive season which likely has a higher trauma prevalence compared to autumn (21%) and winter (13%).

Data were extracted from the WCEMS ePCR system and analysed using the SPSS statistics software (IBM. 2017. SPSS Statistics: Version 25. Armonk, NY: IBM Corps). Shapiro-Wilk tests were conducted to assess for normality. Descriptive statistics (frequency, percentages, median (*M*) and interquartile range (*IQR*)) were calculated for patient characteristics, incident types, injuries sustained, pain score, pain severity and non-pharmacological and pharmacological pain management and presented in graphs and tables. The Pearson chi-square test of independence (inferential statistics) was used to determine relationships between the categorical variables, pain score recorded (yes/no) and age group (≤ 14 and > 14 years), gender, final triage colour (yellow, orange and red) and analgesic medication administrated (yes/no) as well as between analgesic medication administrated (yes/no) and age group (≤ 14 and > 14 years), gender, crew highest qualification (BLS, ILS and all ALS levels) and pain severity (mild, moderate or severe). If relationships between categorical variables were identified, the strength of association was assessed with Phi (*ϕ*) and Cramer’s V (*ϕ*_c_) correlation coefficient.

## Results

### Patient characteristics

Of the 2401 records reviewed, 272 (11.3%) patients were ≤ 14 years of age (*M* = 7, *IQR* = 3–11) while the remaining 2129 (88.7%) were > 14 years (*M* = 31, *IQR* = 24-41) of which 80.5% (*n* = 1713) were between 15–44 years of age. High acuity patients (SATS red or orange) accounted for 35.0% (*n* = 839) of all cases (Table 1).

**Table 1:** Patient characteristics

Characteristics		*n* (%)
Gender		
Male		1650 (68.7%)
Female		751 (31.3%)
Crew highest qualification		
Basic life support (BLS)		415 (17.3%)
Intermediate life support (ILS)		1321 (55.0%)
Advanced life support (ALS)^*a*^		665 (27.7%)
South African Triage Scale (SATS)-priority colour	Time target	
Red	Immediate	200 (8.3%)
Orange	< 10 min	639 (26.6%)
Yellow	< 1 h	1562 (65.1%)
Total		**2401 (100%)**

^*a*^Advanced life support includes the following qualifications: Emergency Care Technician (ECT) (*n* = 299, 12.5%), Paramedic (Critical Care Assistant (CCA) and National Diploma in Emergency Medical Care (NDEMC)) (*n* = 324, 13.5%) and Emergency Care Practitioner (ECP) (*n* = 42, 1.7%)

### Incident types and injury sustained

Assault, transport-related incidents and accidental injuries were the three most common types of incidents (See Additional file 1). The specific injuries sustained were noted in the working diagnosis of 1278 (53.2%) patients with 139 (10.9%) of these sustaining more than one injury (Table 2).

**Table 2:** Specific injuries sustained by patients as documented (*n* = 1278)

Specifics of sustained injuries	≤ 14 Years ***n*** (%)	> 14 Years ***n*** (%)	Total ***n*** (%)
More than 1 injury sustained	10 (0.8%)	129 (10.1%)	139 (10.9%)
Fractures/dislocations/deformities	34 (2.7%)	155 (12.1%)	189 (14.8%)
Burns	21 (1.6%)	38 (3%)	59 (4.6%)
Gunshot wound (GSW)	1 (0.1%)	34 (2.6%)	35 (2.7%)
Polytrauma	0 (0%)	12 (0.9%)	12 (0.9%)
Head injury	18 (1.4%)	102 (8%)	120 (9.4%)
Pneumo-, haemothorax or cardiac tamponade	0 (0%)	25 (2%)	25 (2%)
Sprains/strains/muscle Injuries	1 (0.1%)	11 (0.8%)	12 (0.9%)
Rape	0 (0%)	2 (0.2%)	2 (0.2%)
Neck and/or back pain/tenderness/injury	4 (0.3%)	86 (6.7%)	90 (7%)
Drowning	2 (0.15%)	2 (0.15%)	4 (0.3%)
Open and/or closed wounds	73 (5.7%)	783 (61.3%)	856 (67%)

Since about 11% of patients sustained more than one injury, the total injuries sustained will account to more than 1278.

Almost 15% (*n* = 189) of patients reportedly sustained fractures/dislocations/deformities (with 9 (4.8%) sustaining more than one fracture). The most common injury site was lower extremities (*n* = 78, 41.3%) followed by upper extremities (*n* = 63, 33.3%) while 5 (2.6%) of these patients injured both upper and lower limbs, and 15 (8%) patients were thought to have a pelvis/hip fracture.

Of the 59 (4.6%) patients who had burns documented, 25 (42.4%) had a percentage of burn area recorded (range 1–80%). The remaining patients either had no description of the burn or had the burn described in terms of location, type of burn and/or burn severity.

### Pain score and pain severity

**Fig. 3:**
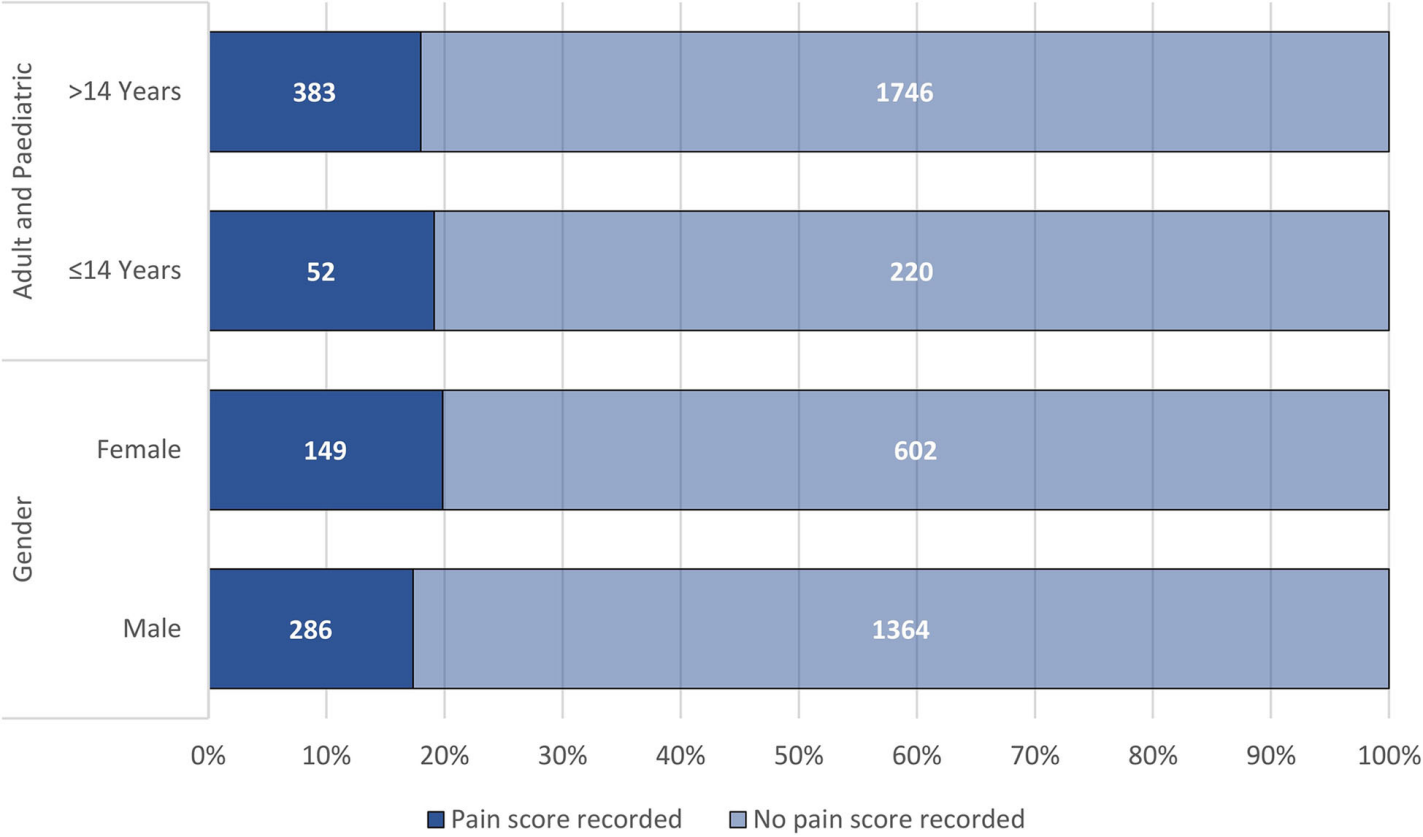
Comparison of pain assessment between gender and age group

A total of 435 (18.1%) patients had a pain score recorded. The median pain score was 6 (*IQR* 4–8). Seventy-two (16.6%) of the patients with a pain score had at least one repeated pain score recorded. Figure 3 illustrates the proportion of records in which a pain score was recorded by gender and age group (adult and paediatric).

**Fig. 4:**
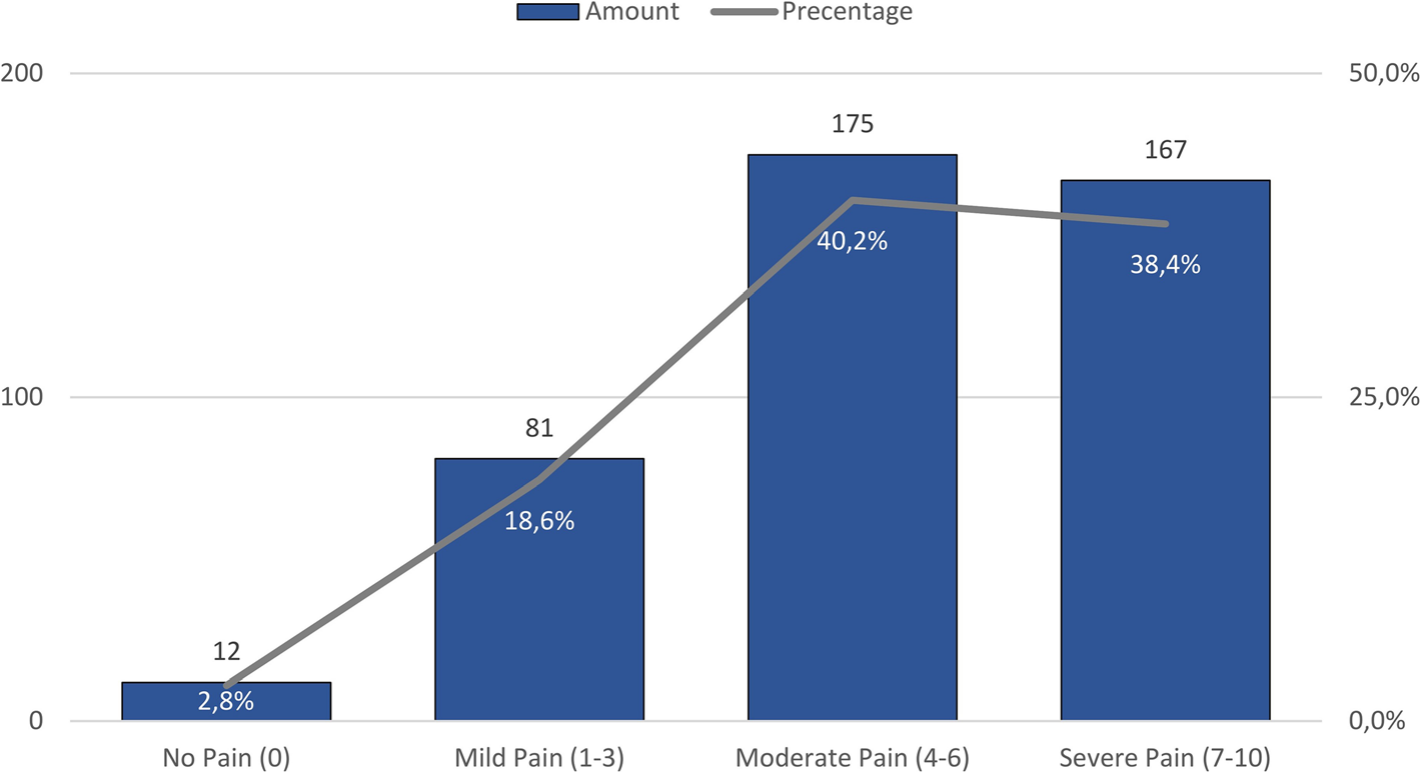
Pain severity of patients with pain score recorded

No association was found between a pain score being recorded and age group (≤ 14 versus > 14 years) (*p* = 0.649), gender (*p* = 0.139) or final triage colour (*p* = 0.076). The majority (78.6%) of those with a pain score reported moderate-to-severe pain (Fig. 4). A further 194 (8.1%) patients had the presence of pain and/or tenderness reported in the working diagnosis, but no pain score recorded. In total, pain was recorded in 617 (*n* = 2401, 25.7%) patients. Of note, the records of numerous other patients indicated injuries likely to be painful for which the presence of pain was not recorded.

### Non-pharmacological pain management

A range of physical non-pharmacological management approaches were recorded in the ePCR including haemorrhage control (*n* = 680, 28.3%), application of splints (*n* = 106, 4.4%) and burn dressings (*n*=57, 2.4%).

### Pharmacological pain management

Only 68 (*n* = 2401, 2.8%) patients received medication with analgesic properties of which 27 (39.7%) had a pain score recorded. IV morphine was administered to 66 (*n* = 68, 97.0%) patients while one received IV ketamine and one intramuscular (IM) diclofenac, firstly or only. Of all the patients (*n* = 66) who received IV morphine, 10 (15.2%) received an additional morphine dose (all adults) and 6 (9.1%) received ketamine [IV or IM] (one paediatric) in addition to the initial morphine. None of the patients received inhaled nitrous oxide.

Of the 342 patients with moderate-to-severe pain recorded, only 7.6% (*n* = 26) received analgesic medication. Fifty-two (78.8%) of the 66 patients who received morphine were male, while 4 (6.1%) were ≤ 14 years. The patients who received ketamine and diclofenac were both > 14 years and male. No association was found between the administration of analgesic medications and gender (*p* = 0.054) or age group (≤ 14 versus > 14 years) (*p* = 0.151).

For six (8.8%) of the patients who received analgesic medication, the highest qualification of the crew was documented as BLS (*n* = 1, 1.5%) or ILS (*n* = 5, 7.3%) while for the remaining 62 (91.2%) patients it was documented as an ALS level qualification (Emergency Care Technician (ECT): *n* = 22, 32.3%, ALS: *n* = 32, 47.1% and Emergency Care Practitioner (ECP): *n* = 8, 11.8%). An association was found between the documented highest qualification (BLS, ILS and all ALS levels) and the administration of analgesic medication (*p* < 0.001). Patients, where the highest qualification was documented as an ALS (ECT, ALS and ECP) level, were more likely to receive analgesia medication. The strength of the association was weak (*ϕ*_*c*_ = 0.242).

Additionally, an association was found between a pain score being recorded and the administration of analgesic medication (*p* < 0.001). Patients with a pain score were more likely to receive analgesic medication; however, the strength of association was weak (*ϕ* = -0.096). An association was also found between pain severity (mild, moderate or severe pain) and the administration of analgesic medication (*p* = 0.001). Patients with severe pain were more likely to receive analgesic medication; however, the strength of association was weak (*ϕ*_*c*_ = 0.188).

## Discussion

To our knowledge, this study is the first to describe the epidemiological characteristics of acute traumatic pain in the African prehospital setting and only the second to describe prehospital pain management practices in the Western Cape Province of South Africa [21].

### Traumatic pain and pain assessment

Our findings indicate that many patients that sustained injuries likely to be painful while only a quarter of patients had pain recorded in some form. Less than a fifth of patients had pain measured with a pain assessment tool with the prevalence of moderate-to-severe pain found to be high (> 75%). International studies, likewise, report the prevalence of pain among trauma patients to be high (> 70%) [8, 31] with a high likelihood of moderate-to-severe pain [5, 9, 11, 12]. This study found pain assessment practices to be poorer than those reported by most international studies [7, 8, 18, 19]. The results, however, are similar (18.1% versus 21%) to pain assessment practices previously found among ALS practitioners in Cape Town, South Africa, with better rates of pain reassessment found in the current study (16.6% versus 6%) [21].

The lack of pain assessment has been identified as a hindrance to adequate pain management [14, 32]. The present study supports these findings as patients with a pain score recorded were more likely to receive medication with analgesic properties. Numerous factors contributing to poor pain assessment documentation have been identified. Being younger, being attended to between 00:00 and 06:00 and shorter transport distances were associated with a reduced likelihood of pain assessment documentation, in children [18]. Adults, in contrast, appear more likely to have pain assessment recorded [33, 34] although this finding is not supported by the current study. Finally, uncooperative patients and communication difficulties have been identified as barriers to pain assessment [18, 35] while the lack of validated age-appropriate pain assessment tools for preverbal children has been identified as a barrier to the management of pain [18].

The use of age-appropriate pain scales as part of general patient care, and regarding all trauma patients with acute pain as candidates for analgesia with regular pain reassessment, is evidence-based recommendations made in a clinical practice guideline (CPG) published in the United States of America [36] and recently adopted for the South African EMS CPGs [37]. Employing observational pain scales is recommended for paediatrics < 4 years [36] and would be more appropriate than the current smiley emoticons found in the ePCR, while the Abbey Pain Scale is a suggested option for the cognitively impaired patient in the prehospital setting [38].

While clear prehospital pain assessment guidelines are helpful, the most frequent reason proposed for the insufficient documentation of pain assessment is a lack of pain knowledge [14, 39, 40]. Several studies have shown that educational activities improve the documentation of pain severity, characteristics and reassessment [41, 42]. In addition to educational activities, EMS systems need to encourage systematic pain assessment and the proper clinical documentation thereof.

### Non-pharmacological pain management practices

Non-pharmacological pain management interventions are more commonly associated with the non-emergency setting. However, cognitive and psychological interventions like reassurance, distraction, and physical interventions like positioning, splinting fractures and burn dressings can all be utilised in the prehospital setting [43]. Psychological interventions sometimes occur inadvertently and are unlikely to be documented in clinical notes. Pain educational initiatives increase awareness and utilisation of non-pharmacological pain interventions in the prehospital setting [41, 42].

Most prehospital studies examine acute pain retrospectively [7, 8, 31] and do not report much, if at all, on non-pharmacological pain management thus limiting comparison. The lack of documentation of non-pharmacological pain management also made an evaluation of these treatments in the current study challenging.

### Pharmacological pain management practices

Morphine, ketamine and diclofenac (not in the scope of South African prehospital practitioners)^1^ were the only medications with analgesic properties administered during this study. Despite the high prevalence of moderate-to-severe pain, less than 8% of those patients, and less than 3% of all the trauma patients received any analgesia. These results are substantially worse than those reported by studies conducted in high-income countries (HIC) although these also reported prehospital pain relief (any aetiologies) to be poor (8 to 42%) [8, 18, 19].

This study revealed that inhaled nitrous oxide was not used. Similar observations were identified by Matthews et al. [21] in the same setting. In the WC, for most emergency care providers (± 84% are operational BLS/ILS) [24], inhaled nitrous oxide is the only prehospital analgesic option. Pain management decision-making for these practitioners is thus limited to requesting the assistance of a higher qualified practitioner (frequent unavailability), non-pharmacological pain management and transportation to a medical facility for further management. The lack of availability of this treatment is a major barrier to effective pain management. Practitioners must have access to analgesic medications as pain care is both a measure of quality emergency care and a human right [44].

Our findings do not suggest disparity between adult and paediatric pain management. This is not consistent with other studies which suggest that adults are more likely to receive opioid analgesics [32, 34, 45]. Further, studies report that women, regardless of age or pain severity, are less likely to receive analgesia [45–48], a finding which was not supported in this study. Our findings suggest, like other evidence, that patients with more severe pain recorded are more likely to receive analgesia [46, 49].

A finding which is difficult to explain was that six patients received medication with analgesic properties from crews not licensed to administer those medications. We attribute this to either documentation errors or in some high workload situations emergency care providers may be transporting patients after an analgesic medication had been administered by a higher qualified practitioner on scene.

### Barriers to pain assessment and management in the prehospital setting

Findings of the study are concerning; however, consideration must be given to the possible reasons for the apparent poor pain assessment and management practices among emergency care providers in the Western Cape, South Africa.

Studies conducted in HIC identified several constraints to prehospital pain assessment and management [32, 35, 40, 50–52]. A barrier commonly highlighted is knowledge deficit, attributed to limited attention to pain assessment and management during initial training and a lack of ongoing education [32, 40, 51, 52]. The lack of alternative routes of medication administration, guideline restrictions or inadequacies, the need to obtain permission and the reluctance of medical control to approve prehospital analgesia administration are, also, previously identified constraints [15, 32, 35, 40, 52]. Further barriers include negative feedback from ED staff or supervisors, organisational culture, scarcity of higher qualified practitioner, absence of monitoring guideline adherence and communication [35, 40, 50, 52].

The prehospital setting is a challenging and dangerous work environment with emergency care providers in South Africa increasingly confronted by the threat of violence. In addition to the concerns for personal safety, high workload and demands on emergency care providers, analgesic agents are only available to a small proportion of prehospital practitioners in South Africa. Research to describe barriers and enablers to prehospital pain assessment and management in the South Africa setting may identify further issues. Epidemiological studies, further investigating inequalities in pain assessment and management, as well as the prevalence, assessment and management of pain in medical and obstetric cases, will add to the knowledge base.

### Study limitations

Like most other observational studies, retrospective reviews have various potential sources of bias including selection and information bias, uncertainty about generalisability and issues with missing data [53]. Probability sampling strategy was used to minimise sampling bias and select a representative sample of the population to allow generalisability of findings [54]. The random selection of the sample from a broader trauma population of the Western Cape, the high burden of trauma and the profile of EMS in the rest of South Africa, mean that the study findings are likely generalisable to prehospital trauma patients in the rest of South Africa. The results may be less generalisable outside South Africa where the burden of trauma may be different, and the profile of EMS systems differ [55]. International studies suggest medical and gynaecological or obstetric conditions to be less common aetiologies of prehospital acute pain [4, 7], and this is another area for further research in low and middle-income settings. Inaccuracies and poor quality of ePCR clinical notes were the foremost limitations to the study findings; however, this may not be a reflection of clinical practice, as an inherent restriction of the retrospective review methodology is the assumption that if it was not documented, it was not done.

## Conclusion

Pain assessment and management were shown to be significantly lacking. Much can be done to improve pain care in the South African prehospital setting. For instance, better pain education during undergraduate studies, ongoing pain education, an EMS culture prioritising pain relief, monitoring pain care quality, optimising resources (most importantly ensuring inhaled analgesia availability), scope of practice revision to consider other analgesic agents suitable for the setting and specifically for BLS and ILS practitioners, specific guideline recommendations for mild, moderate and severe pain and promoting pain assessment, reassessment and redosing to optimise pain care as well as the proper documentation thereof. This study provides clear directions for future research which could further improve pain assessment and management.

## Supplementary information

**Additional file 1:** Types of emergency incidents.

## Data Availability

The datasets used and/or analysed during the study are not publicly available but are available from the corresponding author on reasonable request.

## Abbreviations

ALS: Advanced life support
BLS: Basic life support
CPGs: Clinical practice guideline
ECP: Emergency Care Practitioner
ECT: Emergency Care Technician
ePCRs: Electronic patient care reports
ED: Emergency department
EMS: Emergency Medical Service
HIC: High-income countries
ILS: Intermediate life support
IQR: Interquartile range
IV: Intravenous
IM: Intramuscular
IN: Intranasal
M: Median
SATS: South African Triage Scale
WCEMS: Western Cape Emergency Medical Services

## Footnotes

1 IM Diclofenac was likely administered by a doctor on scene prior to transportation to hospital by ambulance.
