# Supplementary material for: Prehospital acute traumatic pain assessment and management practices in the Western Cape, South Africa: a retrospective review
Source: Int J Emerg Med. 2020 May 5;13:21. doi: 10.1186/s12245-020-00278-w (PMC7201999; doi:10.1186/s12245-020-00278-w)
Supplement: Supplementary file 1 — Additional file 1: Types of emergency incidents. [file 12245_2020_278_MOESM1_ESM.pdf]

Additional File 1: Types of emergency incidents

Incident Type	n (%)
Assault [a]	1211 (50.4%)
Transport-related incident [b]	492 (20.5%)
Accidental injury [c]	380 (15.8%)
Burns [d]	90 (3.8%)
Self-harm	90 (3.8%)
Gunshot wound (GSW)	68 (2.8%)
Environmental	63 (2.6%)
Drowning	4 (0.2%)
Electrocution	3 (0.1%)
**Total**	**2401 (100%)**

Footnote: [a] Includes physical assault, assault with a weapon and sexual assault, [b] Includes light motor vehicle, bus/taxi, truck/heavy vehicle, motorcyclist, cyclist, pedestrian, train, and railway incidents, [c] Includes domestic, sport and industrial accidental injuries, [d] Includes burns and corrosives, residential and informal structure fires.
